# Investigation of the Bacterial Communities Associated with Females of *Lutzomyia* Sand Fly Species from South America

**DOI:** 10.1371/journal.pone.0042531

**Published:** 2012-08-03

**Authors:** Mauricio R. V. Sant’Anna, Alistair C. Darby, Reginaldo P. Brazil, James Montoya-Lerma, Viv M. Dillon, Paul A. Bates, Rod J. Dillon

**Affiliations:** 1 Biomedical and Life Sciences, School of Health and Medicine, Lancaster University, Lancaster, United Kingdom; 2 Institute of Integrative Biology, University of Liverpool, Liverpool, United Kingdom; 3 Laboratório de Bioquímica e Fisiologia de Insetos, Instituto Oswaldo Cruz - Fundação Oswaldo Cruz, Rio de Janeiro, Brasil; 4 Biology Department, Universidad del Valle, Cali, Valle, Colombia; Centro de Pesquisas René Rachou, Brazil

## Abstract

Phlebotomine sand flies are vectors of *Leishmania* that are acquired by the female sand fly during blood feeding on an infected mammal. *Leishmania* parasites develop exclusively in the gut lumen during their residence in the insect before transmission to a suitable host during the next blood feed. Female phlebotomine sand flies are blood feeding insects but their life style of visiting plants as well as animals, and the propensity for larvae to feed on detritus including animal faeces means that the insect host and parasite are exposed to a range of microorganisms. Thus, the sand fly microbiota may interact with the developing *Leishmania* population in the gut. The aim of the study was to investigate and identify the bacterial diversity associated with wild adult female *Lutzomyia* sand flies from different geographical locations in the New World. The bacterial phylotypes recovered from 16S rRNA gene clone libraries obtained from wild caught adult female *Lutzomyia* sand flies were estimated from direct band sequencing after denaturing gradient gel electrophoresis of bacterial 16 rRNA gene fragments. These results confirm that the *Lutzomyia* sand flies contain a limited array of bacterial phylotypes across several divisions. Several potential plant-related bacterial sequences were detected including *Erwinia* sp. and putative *Ralstonia* sp. from two sand fly species sampled from 3 geographically separated regions in Brazil. Identification of putative human pathogens also demonstrated the potential for sand flies to act as vectors of bacterial pathogens of medical importance in addition to their role in *Leishmania* transmission.

## Introduction

Phlebotomine sand flies are responsible for the spread of the medically important *Leishmania* parasites that populate the female sand fly gut. In Brazil *Lutzomyia longipalpis* is the main vector for *Leishmania infantum*, the causative agent of severe visceral leishmaniasis [1]. Visceral Leishmaniasis (VL) in Corumbá, central Brazil [2] is transmitted by *Lu. cruzi*. The macro-ecology of the infection cycle for *Leishmania infantum* is complex; in this case *Lu. longipalpis* is the vector, domestic dogs are the main reservoir hosts in urban environments with humans as the accidental hosts [3]. Very little is known about the microbial ecology of *Lutzomyia* spp. but they are exposed to an unusually diverse range of microbial communities. Although commonly termed ‘blood feeding insects’, adult sand flies are also plant-feeders; only the female requires occasional blood as a protein source for egg production. Both males and females visit plants to acquire carbohydrates where they will also acquire plant phyllosphere microbiota [4] which may be ingested directly into the gut after piercing leaves and stems [5]. The juvenile larval stages feed on animal faeces and plant material that are undergoing microbial biodegradation. Many of the microorganisms ingested by the larvae will be killed during the pupal stage (when the larval gut is reabsorbed) but some may survive pupation to re-colonise the adult gut [6].

Bacteria in other medically important insect vectors may confer a degree of colonisation resistance upon the host insect, reducing parasite populations within the insect’s gut and interfering with disease transmission [7]–[11]. While *Plasmodium* sp. cross through the gut barrier the *Leishmania* parasite is confined to the gut lumen of the female sand fly. The acquired gut bacteria are therefore potentially more important in sand flies because some bacterial species might compete with *Leishmania*.

In communities where there is an epidemic of leishmaniasis the prevalence of *Leishmania* in sand flies seldom exceeds 2% of the female sand fly population [12]–[14]. There are many macro-ecological factors governing the prevalence of sand flies with transmissible infections including the number of mammals with infective leishmaniasis. The presence of a robust resident microbiota within the female sand flies may also impede development of a transmissible parasite population. This would therefore need to be considered when predicting the effect of ecological factors on *Leishmania* transmission. The microbial ecology of phlebotomine sand flies has been the subject of only a few studies [6], [9], [15]–[18] and these have mainly focused on culturable bacteria. One study of cultivatable bacteria associated with the gut of *Lutzomyia longipalpis* [17] included a pre-enrichment step and the identification of isolates by 16S rDNA sequence analysis. The range of species included members of Enterobacteriaceae, such as *Citrobacter, Enterobacter, Serratia, Pantoea, Morganella* and other genera such as *Acinetobacter, Burkholderia, Flavimonas, Pseudomonas* and *Stenotrophomonas*. A bacterial cultivation approach was used to identify bacteria from *Phlebotomus argentipes* in India with potential for paratransgenetic strategy [18]. A comprehensive metagenomic approach done by pyrosequencing analysis from field-caught male and female *L. longipalpis* identified several bacteria from environmental sources, as well as fungi and protists [19]. Characterization based on a non-cultivatable approach is a pre-requisite for understanding the functional role of the microbiota of phlebotomine sand flies in survival and *Leishmania* transmission. The aim of the study was to examine the bacterial biota and potential endosymbionts associated with female *Lu. longipalpis* and *Lu. cruzi* captured in 4 distantly located regions of Brazil and Colombia.

## Results

### Analysis of the 16S Gene Clone Library from Field-collected *Lu. longipalpis* and *Lu. cruzi*

Characterization of bacteria associated with *Lutzomyia* by sequencing a portion of the bacterial 16S rRNA gene from clone libraries led to the identification of 38 bacterial sequences through BLAST searches, with 19 distinct bacterial phylotypes (Table 1). The relative abundance of different phylotypes in the clone libraries was estimated by band pattern comparison of PCR-amplified bacterial 16S rRNA gene fragments derived from single clones run on DGGE, giving a measure of relative abundance for each bacterial species within the gut of the female *Lutzomyia* sand fly (Table 2). Between 26 to 28 clones were analysed from bacterial 16S rRNA clone libraries obtained from 4 pools of field-collected females of *Lu. longipalpis* and *Lu. cruzi*. These wild-caught sand flies were captured from four distantly located regions in Brazil and Colombia. On the basis of sequence similarity to existing Genbank database sequences, the majority of the bacterial sequences obtained from *Lu longipalpis* collected in Lapinha belonged to the Betaproteobacteria class. *Ralstonia* spp. (plant associated species) were the most abundant bacterial phylotype, present in 65.5% of the randomly picked clones (Table 2). The second most frequent bacteria also belong to Betaproteobacteria (*Leptothrix* sp. −17.2%), followed by the Alphaproteobacteria *Bradyrhizobium japonicum* (10.3%), with the Gram positive Firmicutes *Clostridium disporicum* and the Alphaproteobacteria *Caulobacter* sp. being the least abundant in the Lapinha’s field samples (3.4%).

**Table 1:** Bacterial phylotypes associated with *Lutzomyia* sand flies sampled from 4 field sites.

Clone number	Length (bp)	Bacterial Division	Database match (accession no.)	Score Similarity (%)
***Lu. longipalpis*** **, Lapinha**				
1–1	750	Betaproteobacteria	*Ralstonia* sp. (AY216797)	1455 (99)
1–3	781	Betaproteobacteria	*Leptothrix* sp. (AF385528)	1501 (99)
1–4	557	Firmicutes	*Clostridium disporicum* (Y18176)	985 (98)
1–6	851	Betaproteobacteria	*Ralstonia pickettii* (AY268180.1)	1631 (99)
1–9	914	Betaproteobacteria	*Ralstonia* sp. (AY216797)	1741 (99)
1–11	731	Alphaproteobacteria	*Caulobacter* sp. (AY807064.1)	1419 (99)
1–13	620	Alphaproteobacteria	*Bradyrhizobium japonicum* (AB195988)	1229 (100)
1–15	913	Betaproteobacteria	*Ralstonia* sp. (AY216797)	1717 (99)
1–20	918	Betaproteobacteria	*Ralstonia* sp. (AY191853)	1764 (99)
1–23	861	Alphaproteobacteria	*Bradyrhizobium japonicum* (AF208517)	1657 (99)
1–26	913	Betaproteobacteria	*Ralstonia* sp. (AY216797)	1746 (99)
***Lu. longipalpis*** **, Alagoas**				
2–7	898	Actinobacteridae	*Propionibacterium acnes* (AY642051)	1764 (99)
2–8	918	Gammaproteobacteria	*Erwinia billingiae* (AM055711)	1663 (98)
2–11	913	Betaproteobacteria	*Ralstonia* sp. (AY216797)	1762 (100)
2–14	914	Gammaproteobacteria	*Enterobacter* sp. (NJ-1 AM396909)	1616 (98)
2–17	918	Gammaproteobacteria	*Pantoea* sp. (NJ-87 AM419023)	1618 (98)
2–18	892	Actinobacteridae	*Nocardioides albus* (AF004997)	1703 (99)
2–19	914	Betaproteobacteria	*Ralstonia* sp. (AY216797)	1748 (99)
2–23	850	Gammaproteobacteria	*Serratia* sp. (AY827577)	1655 (99)
2–24	909	Gammaproteobacteria	*Klebsiella pneumoniae* (AY043391)	1618 (98)
2–25	913	Gammaproteobacteria	*Acinetobacter* sp. (DQ227342)	1784 (99)
2–27	916	Bacteroidetes	*Sphingobacterium daejeonense* (AB249372)	1536 (96)
2–30	916	Gammaproteobacteria	*Klebsiella pneumoniae* (AY043391)	1659 (97)
***Lu. cruzi*** **, Corumbá**				
3–2	858	Alphaproteobacteria	*Caulobacter* sp. (AY807064)	1427 (99)
3–4	844	Firmicutes	*Clostridium glycolicum* (AY007244)	1481 (97)
3–5	925	Firmicutes; Bacillales	*Staphylococcus xylosus* (D83374)	1731 (99)
3–15	917	Betaproteobacteria	*Ralstonia* sp. (AY216797)	1756 (99)
3–17	945	Firmicutes	*Lactobacillus zymae* (AJ632157)	1699 (98)
3–19	894	Firmicutes	*Clostridium disporicum* (Y18176)	1667 (99)
3–22	913	Betaproteobacteria	*Ralstonia* sp. (AY216797)	1754 (99)
3–24	906	Bacteroidetes Flavobacteria	*Chryseobacterium meningosepticum* (AY468445)	1360 (94)
3–27	402	Alphaproteobacteria	*Bradyrhizobium japonicum* (AB231926)	733 (98)
3–30	907	Bacteroidetes Flavobacteria	*Chryseobacterium meningosepticum* (AY468445)	1340 (93)
***Lu. longipalpis*** **, El Callejón**				
4–1	906	Alphaproteobacteria	*Saccharibacter floricola* (AB110421)	1562 (97)
4–7	919	Gammaproteobacteria	*Stenotrophomonas maltophilia* (AJ293470)	1570 (99)
4–13	907	Actinobacteria	*Propionibacterium acnes* (AB041617)	1756 (99)
4–18	907	Alphaproteobacteria	*Saccharibacter floricola* (AB110421)	1566 (98)
4–22	898	Bacteroidetes	*Dyadobacter ginsengisoli* (AB245369)	1532 (97)

Insects collected in field locations were suspended in 70% ethanol. DNA extracts from pooled samples of insects were subjected to PCR using primers specific for a 900 bp region of the 16S rRNA gene. The PCR products were cloned and colonies randomly selected. Purified plasmids from the clones were subjected to specific PCR, the products separated with DGGE and representative clones were selected on the basis of the band pattern of their DGGE profiles.

**Table 2:** Abundance of bacterial phylotypes recovered from 16S rRNA gene clone libraries constructed from adult *Lutzomyia* sand flies.

Bacterial Division	Database Match	Abundance (%)
***Lu. longipalpis*** **, Lapinha n = 29**		
Betaproteobacteria	*Ralstonia* sp. AY216797	65.5
Betaproteobacteria	*Leptothrix* sp.	17.2
Alphaproteobacteria	*Bradyrhizobium japonicum*	10.3
Firmicutes	*Clostridium disporicum*	3.4
Alphaproteobacteria	*Caulobacter* sp.	3.4
***Lu. longipalpis*** **, Alagoas n = 28**		
Betaproteobacteria	*Ralstonia sp*. AY216797	50.0
Gammaproteobacteria	*Erwinia billingiae* AM055711	17.8
Gammaproteobacteria	*Klebsiella pneumoniae* AY043391	14.2
Actinobacteridae	*Nocardioides albus* AF004997	3.6
Gammaproteobacteria	*Serratia* sp. AY827577	3.6
Gammaproteobacteria	*Acinetobacter* sp. DQ227342	3.6
Bacteroidetes	*Sphingobacterium daejeonense*	3.6
Actinobacteridae	*Propionibacterium acnes* AY642051	3.6
***Lu. cruzi*** **, Corumbá n = 26**		
Betaproteobacteria	*Ralstonia* sp. AY216797	40.7
Firmicutes; Bacillales	*Staphylococcus xylosus* D83374	25.9
Bacteroidetes	*Chryseobacterium meningosepticum*	11.1
Alphaproteobacteria	*Caulobacter* sp. AY807064	7.4
Firmicutes	*Clostridium* sp.	7.4
Firmicutes	*Lactobacillus zymae* AJ632157	3.7
Alphaproteobacteria	*Bradyrhizobium japonicum* AB231926	3.7
***Lu. longipalpis*** **, El Callejón n = 28**		
Alphaproteobacteria	*Saccharibacter floricola* AB110421	89.2
Gammaproteobacteria	*Stenotrophomonas maltophilia* AJ293470	4.0
Actinobacteridae	*Propionibacterium acnes* AB041617	4.0
Bacteroidetes Sphingobacteria	*Dyadobacter ginsengisoli* AB245369	4.0

Between 26 and 28 clones were randomly selected each of the 4 pools of samples. The PCR products from GC clamped primers [44] were run on DGGE and clones were selected for sequencing on the basis of the gel profile. Gel profiles were grouped according to identical band distribution.

The 16S rRNA gene sequencing data derived from *Lu. longipalpis* collected in Alagoas state (Brazil) showed that the majority of bacterial species identified belonged to Gammaproteobacteria (Table 1). However, BLAST searches confirmed that clones from this library were also dominated by sequences related to the genus *Ralstonia* (50%). The most abundant Gammaproteobacteria were *Erwinia billingiae* (another well known plant pathogen) (17.8%) and *Klebsiella pneumoniae* (14.2%). *Notocardioides albus*, *Serratia* sp., *Acinetobacter* sp., *Sphingobacterium daejeonense* and *Propionibacterium acnes* were identified in 3.6% of the bacterial clones.

The 16S rRNA sequence analysis of *Lu. cruzi* from Corumbá (Mato Grosso – Brazil) identified 8 bacterial genera across 5 different divisions. Again, *Ralstonia* spp. was the most abundant phylotype (40.7%), with the Gram-positive *Staphyloccoccus xylosus* and *Cryseobacterium meningosepticum* being identified in 25.9 and 11.1% of the clones, respectively. The *Caulobacter* sp. (Alphaproteobacteria) and the Gram-positive *Clostridium* sp. (Firmicutes) were found in 7.4% of the individual clones analysed and the Gram-positive bacteria *Lactobacillus zymae* and the *Bradyrhizobium japonicum* (Alphaproteobacteria) were detected in 3.7% of the bacterial clones screened.

The vast majority of the bacterial phylotypes identified from *Lu. longipalpis* collected from cattle in Callejón (Colombia) belonged to the Alphaproteobacteria class: *Saccharibacter floricola* was present in 89.2% of the clones. *Stenotrophomonas floricola* (Gammaproteobacteria), *Propionibacterium acnes* (Actinobacteria) and *Dyadobacter ginsengisoli* (Bacterioidetes) were present in 4% of the bacterial samples analysed.

### Neighbour-joining Phylogram of Bacterial 16S Sequences Obtained from *Lu. longipalpis* Collected from the Field

**Figure 1:**
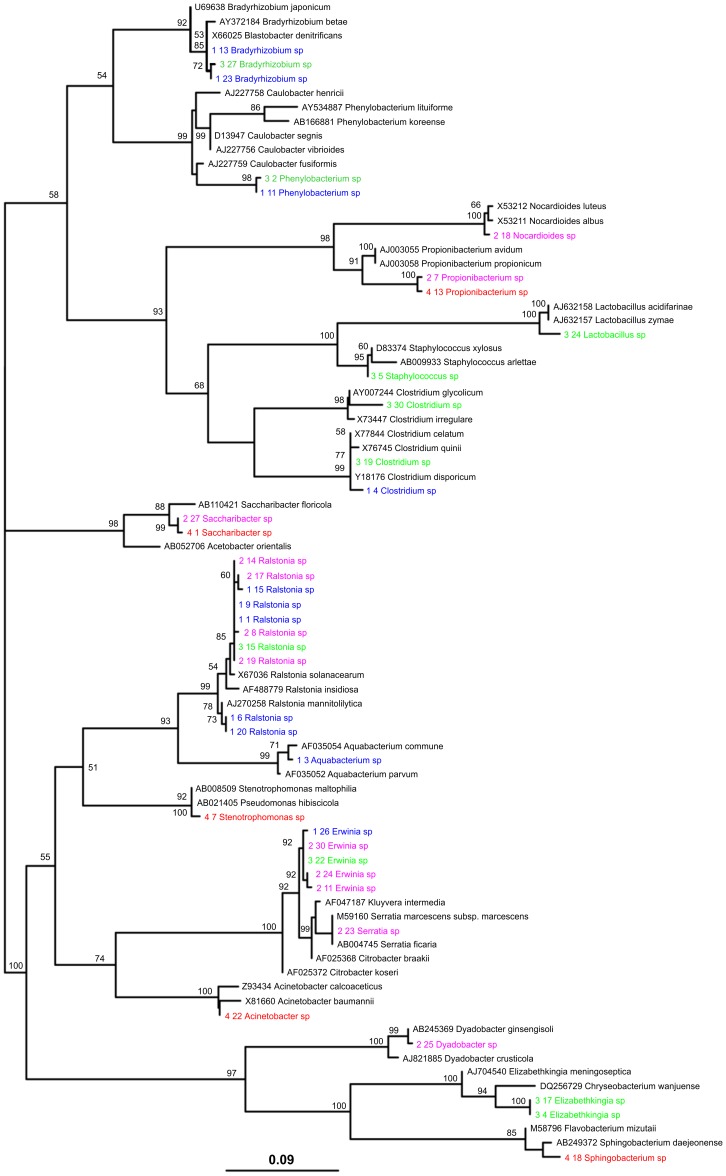
Neighbour-joining phylogram of bacterial 16S sequences obtained from field collected *Lutzomyia* species. Numbering refers to clones described in Table 1. Bacterial species from Lapinha (Minas Gerais-Brazil) are presented in purple; species identified from sand flies collected in Alagoas (Brazil) are presented in green; species identified in Corumbá (Brazil) are presented in pink and red represents bacterial species identified from *Lu. longipalpis* collected in Callejón (Colombia). Bacterial sequences extracted from Genbank for comparison are presented in black.

The 16S gene sequences generated from the clone libraries from *Lu. longipalpis* collected from the field were used to construct a neighbour-joining tree. The topology of the ML and NJ trees (Fig. 1) were identical and showed good correspondence with the RDP classifier results. The phylogenetic analysis showed robust clustering of bacterial species isolated from the different sand fly species and different geographic locations. This is particularly true for the *Ralstonia* and *Erwinia* isolates that showed both robust clades and a high degree of similarity (70% bootstrap support) despite the fact that they were from different sand fly species and from different geographical locations.

### DGGE Profiles of Field Caught *Lu. longipalpis* and *Lu. cruzi*

**Figure 2:**
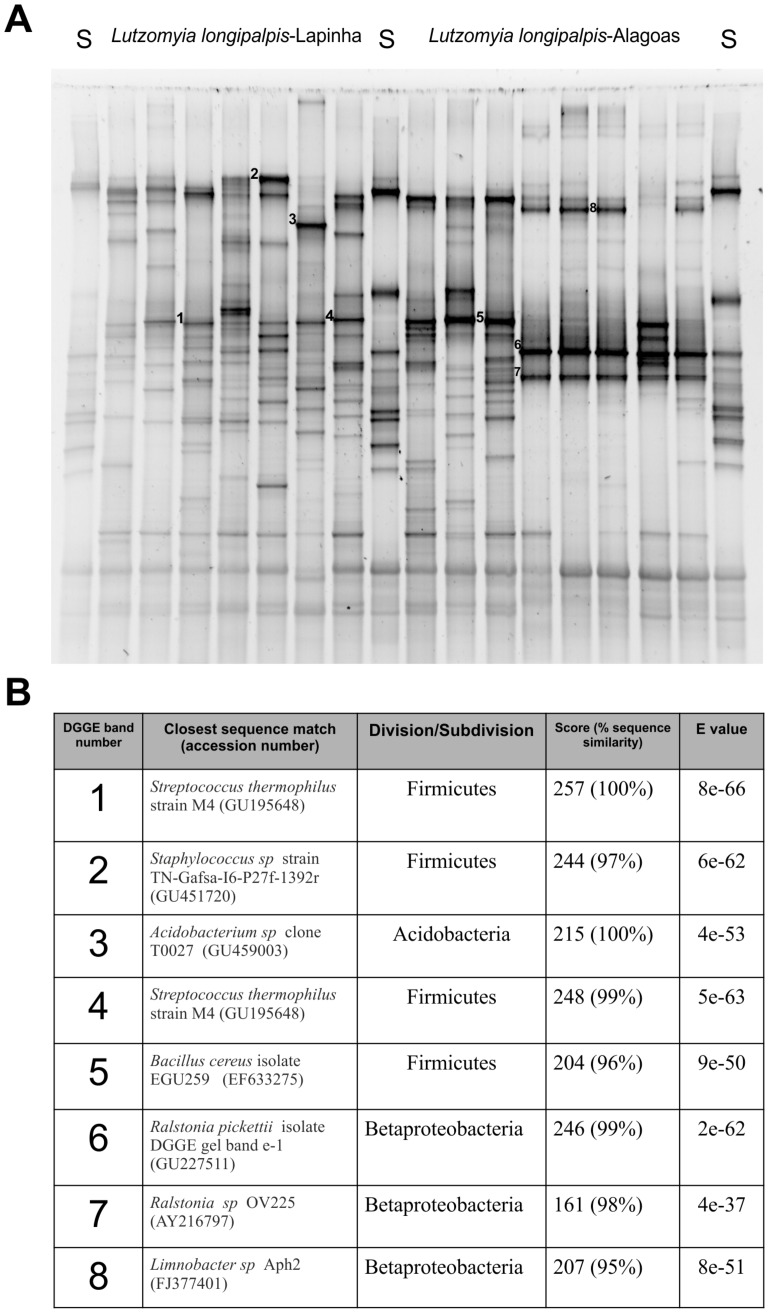
Denaturing gradient gel electrophoresis (DGGE) profiles of PCR-amplified bacterial gene fragments derived from field-collected individual female *Lu. longipalpis.* Lanes L1 to L10 corresponds to DGGE profiles of *Lu. longipalpis* collected from Lapinha cave-Minas Gerais; A1 to A5 correspond to DGGE profiles of *Lu. longipalpis* collected from Alagoas state/Brazil. Lanes labelled S correspond to standard DGGE markers prepared from a selection of bacterial 16S rRNA gene products to enable gel to gel comparison.

**Figure 3:**
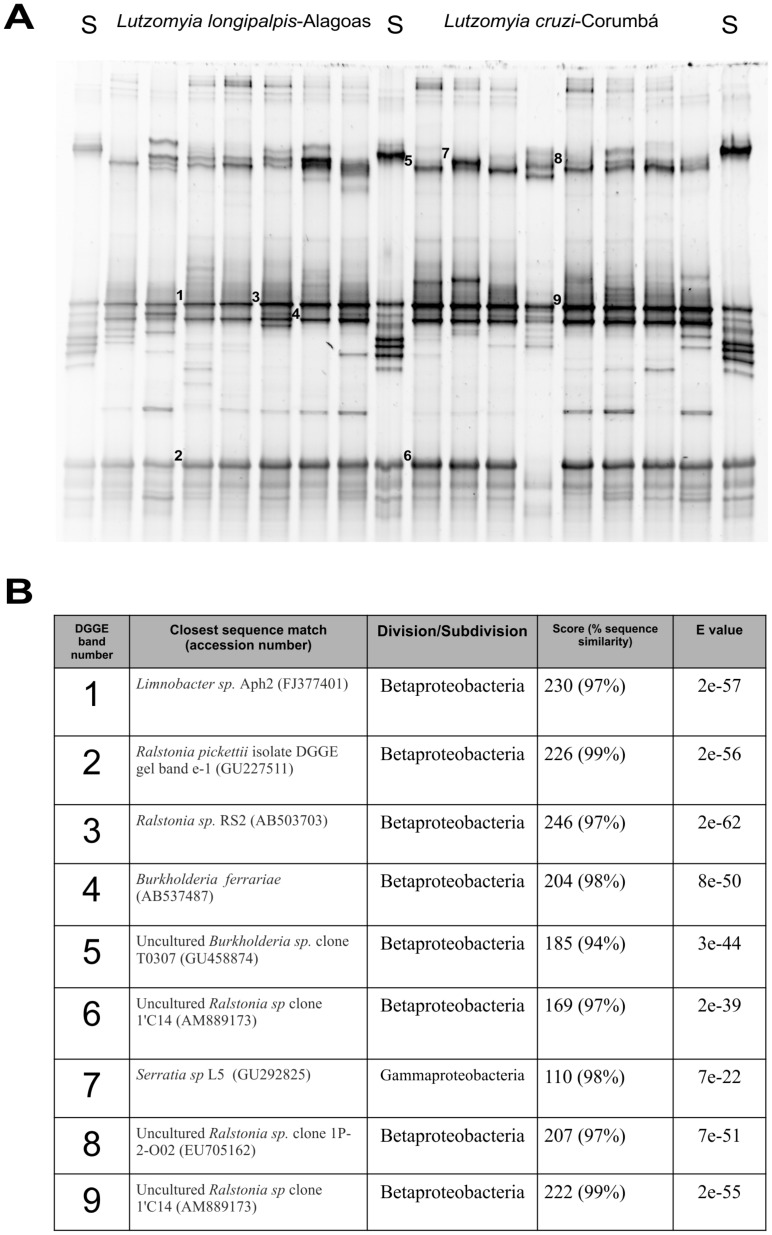
Denaturing gradient gel electrophoresis (DGGE) profiles of PCR-amplified bacterial gene fragments derived from field-collected individual female *Lu. longipalpis.* Lanes A6 to A12 corresponds to DGGE profiles of *Lu. longipalpis* collected from Alagoas state/Brazil; C1 to C8 correspond to DGGE profiles of *Lu. cruzi* collected from Corumbá-Mato Grosso-Brazil. Lanes labelled S corresponds to DGGE markers prepared from a selection of bacterial 16S rRNA gene products to enable gel-to-gel comparison.

Bacterial DGGE profiles were generated using PCR-amplified bacterial 16S rRNA gene fragments from individual female *Lu. longipalpis* collected in Lapinha cave and Alagoas and *Lu. cruzi* collected in Corumbá, Mato Grosso do Sul (Brazil). DGGE band profiles indicated the presence of several putative bacterial phylotypes in *Lu. longipalpis* from different geographic populations and *Lu. cruzi* from Corumbá (Fig. 2 and 3). Random sequencing of DGGE bands identified bacterial species that were not previously detected in our clone libraries for *Lu. longipalpis* from Lapinha cave and Alagoas. DGGE band sequencing also confirmed the presence of *Ralstonia* in individual *Lu. longipalpis* from Alagoas and *Lu. cruzi* from Corumbá (Brazil). It is possible that some bands at identical positions may not be homologous and the diversity of phylotypes may be higher than the profile suggests.

## Discussion

The present study used DGGE and 16S rRNA sequence analysis to identify a relatively low range of bacterial phylotypes across several divisions within female adult phlebotomine sand flies. Other insects such as cockroaches, termites and crickets have a much more complex microbiota reflecting their greater contribution to digestion [20]. Studies using classical microbiological techniques demonstrated that bacteria can be frequently isolated from the gut of female *Phlebotomus* [9] and *Lutzomyia* [15]–[17]. The approach of cultivating the bacteria prior to identification, however, may have introduced an unknown bias into the sampling as the uncultivated bacteria will not have been considered. Dillon et al. [9] isolated bacteria from the midgut of field-collected *P. papatasi* caught in Egypt and found that in the sand flies with blood in their midguts, 59% contained bacteria and some of them contained potential human pathogens. Hillesland et al. [18] identified 28 distinct gut bacteria from *Phlebotomus argentipes*, with *Staphyloccoccus* and members of the family Enterobacteriaceae being isolated in the greatest abundance. In *Lu. longipalpis*, a previous study using 3 Brazilian populations identified *Serratia marcescens*, *Pseudomonas aeruginosa* and *Pantoea agglomerans* as the most frequent bacteria associated with all field populations [17]. The present study was an attempt to investigate the microbiota associated with sand flies of the genus *Lutzomyia* using a molecular approach to identification. A metagenomic analysis of all taxa associated with adult *Lutzomyia longipalpis* from Brazil identified only ten different bacterial types including sequences for *Ralstonia pickettii*, *Anoxybacillus flavithermus*, *Geobacillus kaustophilus*, *Streptomyces coelicolor*, *Propionibacterium acnes*, *Acinetobacter baumannii* and *Veillonella* sp. [19].

The phylotypes in this study were similar to those found in an apparently disparate range of insect species representing different orders of insects. This includes Orthoptera (locusts [21]), Lepidoptera (cabbage white butterfly [22]), Diptera (mosquitoes [23] and tephritid fruit flies [24]). We suggest that they represent insects with low diversity, facultative microbiota that are drawn from the reservoir of phylotypes in the local environment of the insect. It should be noted that the identifications are tentative and some clones may represent novel phylotypes. The relative abundances of different phylotypes in the clone libraries give a measure of relative abundance in the female *Lutzomyia* sand fly. However, larger sample numbers may provide a more accurate comparison of relative abundance.

A notable observation was the apparent abundance of putative *Ralstonia* spp. from 2 sand fly species sampled from 3 geographically separated regions. The association of *Ralstonia* sp. with *Lu. longipalpis* was also found in a previous metagenomic analysis of all taxa associated with this sand fly species from one of the same locations, Lapinha, in Brazil [19]. The most common species is *Ralstonia solanacearum,* the causal agent of bacterial wilt in solanaceous crops. *R. solanacearum* is a soil-borne bacterium originating from the tropics, subtropics, and warm temperate regions [25]. Another potential plant pathogen genus *Erwinia* was also noted. In addition to their plant visiting habits [26]–[28], the female sand flies deposit eggs in terrestrial habitats and the larvae feed on either decaying vegetation or animal faeces. Therefore, there is ample opportunity for these insects to come into contact with plant pathogens. The discovery of *Ralstonia* sp. in our sand fly samples raises the question whether Phlebotomine sand flies may act as vectors of plant pathogens (see also [19]). Future experiments will establish whether sand flies can harbour or mechanically transmit pathogens from a diseased plant to a healthy individual.

The identification of the bacterial sequences from female sand flies with similarity to *Bradyrhizobium* sp. (a bacteria found intracellularly in root nodules) is notable, although a number of nitrogen fixing bacteria have been associated with insects and blood feeding invertebrates such as leeches where a new lineage of α-proteobacterial endosymbionts, related to Rhizobiaceae, have been identified [29]. Is there a role for nitrogen fixing bacteria in sand flies that are unable to find a bloodmeal? There are large populations of diazotrophic enterobacteria that express dinitrogen reductase within the gut of fruit flies (*Ceratitis capitata*) and nitrogen fixation may significantly contribute to the fruit fly nitrogen intake [24], [30]. When a blood source is scarce, the presence of nitrogen fixing bacteria may also be beneficial for the female sand fly. *Chryseobacterium meningosepticum* is an emerging pathogen, an agent of neonatal meningitis and is involved to a lesser extent in cases of pneumonia and bacterial sepsis in neonates and adults [31], [32]. The preliminary identification of this bacterial species in *Lu. cruzi* from Corumbá (Brazil) highlights the potential for identifying sand flies as vectors of bacterial pathogens of medical importance. Other potential human and plant pathogens identified in our field samples are bacterial clones with sequence similarity to genus *Burkholderia* (soil-borne, Gram-negative, motile, obligately aerobic, rod-shaped bacteria). *Burkholderia* has been previously identified from the digestive tract microbiota in female *Lutzomyia longipalpis* [17]. *Burkholderia mallei* is the causative agent of the predominately equine disease glanders and is very closely related to the much more diverse species *Burkholderia pseudomallei*, an opportunistic human pathogen and the primary cause of melioidosis [33].

The nested PCR screening of the sand fly microbiota did not result in identification of endosymbionts *Wolbachia* or *Cardinium* despite successful positive results from control insect material known to contain these species (results not shown). The absence of *Wolbachia* endosymbionts in most *Lutzomyia* species [34] including our own colony raises the question as to whether there are other, as yet, undescribed endosymbionts in these insects. One study [6] has shown that *Ochrobactrum intermedium* survives pupation in *Lu. longipalpis* to appear in the adult gut. This species is also present throughout our *Lu. longipalpis* 25 year old colony originally from Jacobina (Bahia-Brazil). Some of the above mentioned phylotypes could represent endosymbionts. The negative result of *Wolbachia* specific PCR probably means that *Wolbachia* does not naturally infect *Lutzomyia* and this is the first time that the sand fly genus has been tested for *Cardinium*. The lack of these reproductive symbionts in the system may be valuable for the design and deployment of symbiont control strategies. It is also interesting as the lack of these bacteria may suggest either a barrier to their invasion of sand flies or a mismatch in symbiont/host biology.

The molecular microbial ecology approach has resulted in an upsurge in understanding about the relationships of insects with microorganisms; the importance of gut microbes is gaining increased recognition [20], [35]–[38]. Of particular relevance is the hypothesis that resident bacteria of insects may prevent the development of insect pathogens in the gut either directly [7] or indirectly by regulating the host immune response [11]. Adler and Theodor [39] were the first to suggest that the presence of commensal microorganisms might impair *Leishmania* development in the sand fly midgut. Schlein et al. [40] observed yeast-like fungi in lab reared and field caught *Phlebotomus papatasi* and *P. tobbi* and suggested that gut contamination might interfere with *Leishmania* transmission. Only approximately 1.5% of the adult female population contain *Leishmania* parasites in the gut [14] and not all these insects may contain parasites at the mammalian infective (metacyclic) stage. The reasons for the low number of *Leishmania* infected sand flies are related to a number of factors; the main factors being the availability of infected mammalian reservoirs and the short life of the adult sand fly. The presence of competing microbes in the sand flies’ gut could contribute and perhaps reduce even further the chances of a sand fly becoming infected with *Leishmania*.

## Materials and Methods

### Insect Collection Sites

Wild caught *Lu. longipalpis* collected in Brazil were obtained from 3 different localities separated from each other by over 2,000 km: sand flies captured in Estrela de Alagoas (a town located in a rural area of Alagoas State-Brazil) were collected using CDC light traps near the livestock with the presence of adjacent fruit trees; Sand flies collected in Lapinha cave (situated in the municipality of Lagoa Santa-Minas Gerais, Brazil) were collected using a CDC light trap placed near a caged chicken; *Lutzomyia cruzi* were collected in Mato Grosso do Sul State- Corumbá (Brazil) from a rural area next to a pigpen in the backyard of a small house. *Lu. longipalpis* from Colombia came from the small rural community of El Callejón (municipality of Ricaute) and were collected by aspiration from cattle.

### Ethics Statement

No specific permits were required for the insect collections. Specific permission was obtained from the private owners to collect the sand flies from all sites apart from Lapinha where RPB has a long standing agreement to collect sand flies. The field studies did not involve endangered or protected species.

### Extraction and Purification of DNA

Insects were surface sterilized by dipping in 70% ethanol for 30 seconds and then washed twice in sterile saline (NaCl 0.15 M) followed by storage in 90% ethanol. Whole insects were suspended in homogenisation buffer with lysing matrix. Homogenisation was done using a bead beater (Fastprep instrument, MP Biomedicals) for 40 sec. DNA extraction was carried out using the FastDNA Spin Kit for soil (MP Biomedicals) according to the manufacturer’s instructions with the exception that material was centrifuged for 8 minutes at 14,000 g following cell lysis before transferring the supernatant to a clean tube with protein precipitation solution [41]. DNA was stored in a 50 µl aliquot of DNase/pyrogen free water at –70°C. In addition, DNA template was also prepared from pure bacterial cultures as described previously [42]. Essentially, 1 ml of log phase culture was centrifuged for 4 min at 14,000x g. Supernatant was discarded and the cell pellet was re-suspended in 100 µl of 5% (w/v) Chelex 100 (Sigma-Aldrich). The suspension was then heated at 100°C for 5 min prior to placing in ice for 5 min followed by a further heating and cooling step. The crude DNA lysate was then centrifuged as above and used directly for PCR amplification.

### *Lutzomyia* Bacterial Clone Library Production and Sequencing

Partial sequences of the 16S rRNA gene were amplified from DNA extracts from pooled samples of insects (n = 20) using primers 27F and 1492R [43]. Four clone libraries (Lapinha, Alagoas, Corumba, El Callejón) were derived from the amplified products using a cloning kit (TOPO TA, Invitrogen, CA, USA). Purified plasmids from randomly selected clones (n = 26–29) were subjected to DGGE (denaturing gradient gel electrophoresis) specific PCR and the products were run on DGGE (data not shown), representative clones were selected for sequencing on the basis of their DGGE profiles and the abundance of the putative phylotypes estimated on the basis of identical band profiles in comparison to the sequenced clones. Bacterial sequences obtained during this study were deposited at EMBL as HE611532-69.

### DGGE Analysis of PCR Amplified Products from Individual Females

For the DGGE approach amplification of the 16S rRNA genes of bacteria were performed using PCR-DGGE primers 357FGC-518R [44] Amplifications were carried out with 4 pmol ul^−1^ primers, 1 µl of DNA template, 1 x reaction buffer (Promega), 1.5 mM MgCl_2_, 1.5U Taq DNA polymerase (Promega), 0.25 mM each dNTP in a 50 µl PCR reaction mixture with molecular grade water. Positive (pure culture bacterial DNA) and negative controls (water) were routinely included. PCR conditions were 95°C for 5 mins, 10 cycles of 94°C/30 s; 55°C/30 s; 72°C/60 s, 25cycles of 92°C/30 s; 52°C/30 s; 72°C/60 s, followed by 10 min at 72°C. DGGE was conducted according to previously described methods [21], [45]. DGGE marker was prepared from a selection of bacterial 16S rRNA gene products to enable gel to gel comparison. PCR products prepared from single females were separated (ca 200 ng of each product) using a combined polyacrylamide and denaturant gradient between 6% acrylamide/30% denaturant and 12% acrylamide/60% denaturant. A 100% denaturing condition is equivalent to 7 M urea and 40% (v/v) formamide. Gels were poured with the aid of a 50 ml gradient mixer (Fisher Scientific,) and electrophoresis run at 200 V for 5 h at 60°C. Polyacrylamide gels were stained with SYBRGold nucleic acid gel stain (Molecular Probes) for 30 min and viewed under UV.

Bands were excised with sterile razor blades immediately after staining and visualisation of the gels. Gel bands were stored at –70°C, washed with 100 µl distilled water and DNA extracted with 10–20 µl of water depending on band intensity. DNA was re-amplified using the PCR-DGGE primers and products checked by agarose gel electrophoresis. The PCR products were purified using the QIAGEN PCR purification kit (Qiagen Ltd). The products were directly sequenced with the 518R primer. Partial bacterial 16S rRNA gene sequences (approximately 160 bp) were subjected to a NCBI nucleotide blast search (http://blast.ncbi.nlm.nih.gov/Blast.cgi) to identify sequences of the highest similarity.

### Endosymbiont Analysis

Bacterial 16S rDNA was amplified by PCR. The PCR reagents comprised 2 mM MgCl_2_, 0.5 mM each dNTP, 0.5 µM each primer, PCR buffer and 1 U Taq polymerase (Promega, Southampton, UK), and the reaction conditions were 24 cycles of 94°C for 1 min, primer pair annealing temperature for 1 min, 72°C for 1 min, but with the extension time increased to 2 min and 8 min for the first and last cycle, respectively. Universal bacterial 16S rDNA primers were used together with primers specific to *Wolbachia* 16S rRNA gene; primers 99F and 994R [46] and *Cardinium* with related *Bacteroidetes* primers, (forward) Ch-F 5′-TACTGTAAGAATAAGCACCGGC-3′ and (reverse) Ch-R 5′-GTGGATCACTT AACGCTTTCG-3′ [47].

Nested PCR reactions used specific primers and 1 µl cleaned PCR product (QIAquick PCR purification kit, Qiagen, Crawley, UK) from a PCR amplification with the universal bacterial primers and total fly DNA template. All PCR reactions included template-free samples as negative control. PCR products were separated by electrophoresis in a 1.6% agarose gel and visualized under UV following staining with ethidium bromide. Sequences were checked for chimeric artefacts using the CHECK_CHIMERA program of Ribosomal Database Project II (RDP II) [48] and compared with similar rDNA sequences in the DNA databases using the BLAST search program of the National Centre for Biotechnology Information (NCBI) and RDP classifier [49].

For the phylogenetic analysis the Muscle alignment program [50] was used to generate multiple sequence alignment (which was adjusted manually). The neighbour-joining (DnaDist-Neighbour) [51] and maximum likelihood (PhyML) [52] analysis, gaps and ambiguous bases were excluded from the analysis using Gblock [53]. The two methods of tree drawing neighbour-joining (NJ) used the Kimura two-parameter model with correction for multiple substitutions and maximum likelihood (ML) using the HKY85 model that included an estimate of 0.49 for the proportion of sites assumed to be invariable and a transition/transversion ratio of 1.7 (estimated from the data). Bootstrap analyses with 100 replications were performed to estimate the reliability of the resulting gene phylogenies.
